# Crystal structure of *N*-(1-acetyl-3-chloro-1*H*-indazol-6-yl)-4-meth­oxy­benzene­sulfonamide

**DOI:** 10.1107/S2056989015020605

**Published:** 2015-11-04

**Authors:** Yassine Hakmaoui, El Mostapha Rakib, Ahmed Gamouh, Mohamed Saadi, Lahcen El Ammari

**Affiliations:** a Laboratoire de Chimie Organique et Analytique, Université Sultan Moulay Slimane, Faculté des Sciences et Techniques, Béni-Mellal, BP 523, Morocco; b Laboratoire de Chimie du Solide Appliquée, Faculté des Sciences, Université Mohammed V, Avenue Ibn Battouta, BP 1014, Rabat, Morocco

**Keywords:** crystal structure, hydrogen bonding, sulfonamide

## Abstract

In the title compound, C_16_H_14_ClN_3_O_4_S, the six-membered ring of the indazole group is connected to a sulfonamide group. The indazole system is essentially planar, with the greatest deviation from the mean plane being 0.007 (2) Å. The dihedral angle between the two six-membered rings is 74.99 (9)°. The crystal structure exhibits inversion dimers in which mol­ecules are linked by pairs of N—H⋯O and C—H⋯O hydrogen bonds.

## Related literature   

For biological activities of indazole derivatives, see: Gaikwad *et al.* (2015 ▸); Jennings & Tennant (2007 ▸). For related derivatives, see: Abbassi *et al.* (2012 ▸, 2014 ▸); Bouissane *et al.* (2006 ▸).
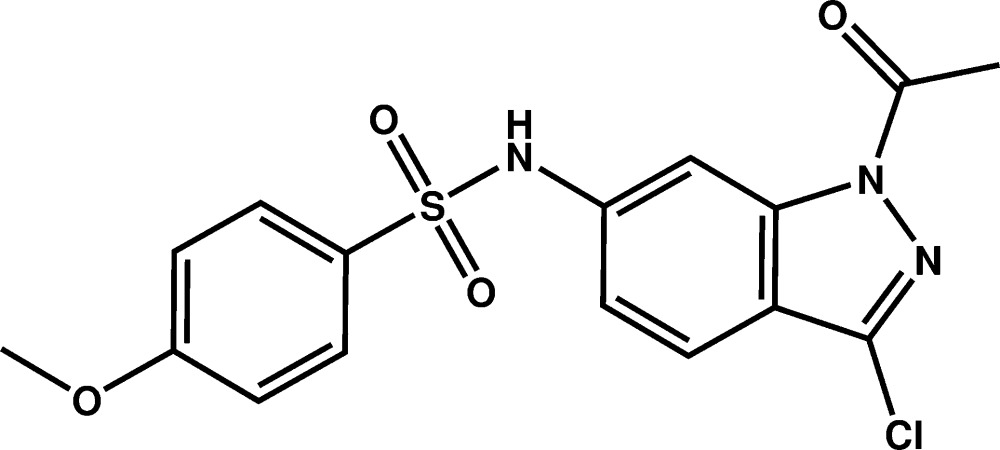


## Experimental   

### Crystal data  

C_16_H_14_ClN_3_O_4_S
*M* *_r_* = 379.81
Monoclinic, 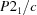

*a* = 13.9664 (6) Å
*b* = 6.4300 (3) Å
*c* = 19.6155 (9) Å
β = 107.227 (1)°
*V* = 1682.52 (13) Å^3^
*Z* = 4
Mo *K*α radiation
μ = 0.38 mm^−1^
*T* = 296 K
0.31 × 0.27 × 0.21 mm

### Data collection  

Bruker X8 APEX diffractometer
Absorption correction: multi-scan (*SADABS*; Bruker, 2009 ▸) *T*_min_ = 0.654, *T*_max_ = 0.747
33806 measured reflections
4461 independent reflections
3423 reflections with *I* > 2σ(*I*)
*R*_int_ = 0.032

### Refinement  

*R*[*F* ^2^ > 2σ(*F* ^2^)] = 0.042
*wR*(*F* ^2^) = 0.130
*S* = 1.09
4461 reflections
227 parameters
H-atom parameters constrained
Δρ_max_ = 0.34 e Å^−3^
Δρ_min_ = −0.35 e Å^−3^

### 

Data collection: *APEX2* (Bruker, 2009 ▸); cell refinement: *SAINT* (Bruker, 2009 ▸); data reduction: *SAINT*; program(s) used to solve structure: *SHELXS97* (Sheldrick, 2008 ▸); program(s) used to refine structure: *SHELXL2014* (Sheldrick, 2015 ▸); molecular graphics: *ORTEPIII* (Burnett & Johnson, 1996 ▸) and *ORTEP-3 for Windows* (Farrugia, 2012 ▸); software used to prepare material for publication: *publCIF* (Westrip, 2010 ▸).

## Supplementary Material

Crystal structure: contains datablock(s) I. DOI: 10.1107/S2056989015020605/tk5402sup1.cif

Structure factors: contains datablock(s) I. DOI: 10.1107/S2056989015020605/tk5402Isup2.hkl

Supporting information file. DOI: 10.1107/S2056989015020605/tk5402Isup3.cml

**Fig. 1.**
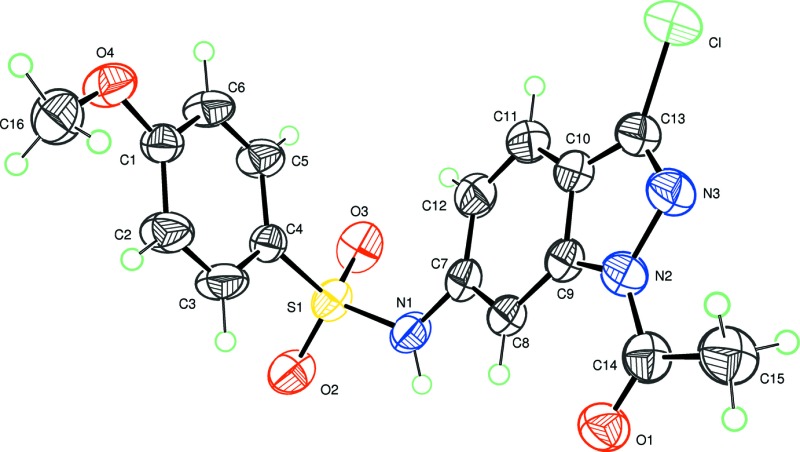
Plot of the molecule of the title compound with the atom-labelling scheme. Displacement ellipsoids are drawn at the 50% probability level. H atoms are represented as small circles.

. DOI: 10.1107/S2056989015020605/tk5402fig1.tif
Plot of the mol­ecule of the title compound with the atom-labelling scheme. Displacement ellipsoids are drawn at the 50% probability level. H atoms are represented as small circles.

**Fig. 2.**
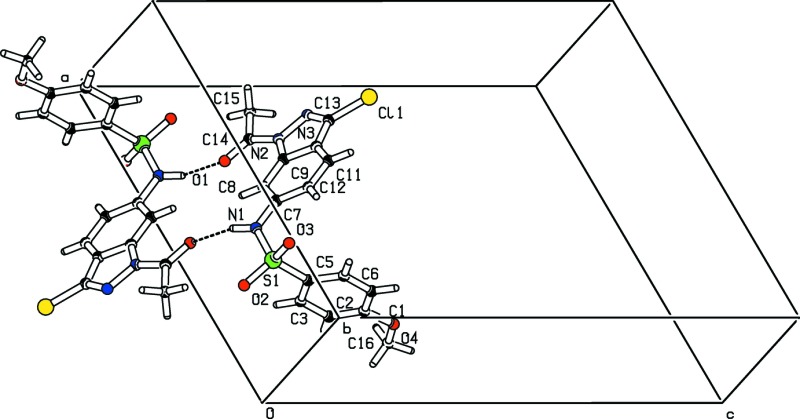
Partial plot of the molecular packing in the title compound, showing inversion dimers of molecules linked through N1—H1···O1 (dashed lines) and C15—H15C···O2 hydrogen bonds.

C . DOI: 10.1107/S2056989015020605/tk5402fig2.tif
Partial plot of the mol­ecular packing in the title compound, showing inversion dimers of mol­ecules linked through N1—H1⋯O1 (dashed lines) and C15—H15*C*⋯O2 hydrogen bonds.

CCDC reference: 1434410

Additional supporting information:  crystallographic information; 3D view; checkCIF report

## Figures and Tables

**Table 1:** Hydrogen-bond geometry (Å, °)

*D*—H⋯*A*	*D*—H	H⋯*A*	*D*⋯*A*	*D*—H⋯*A*
N1—H1⋯O1^i^	0.86	2.19	2.934 (2)	144
C15—H15*C*⋯O2^i^	0.96	2.33	3.251 (3)	159

Symmetry code: (i) 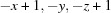.

## supplementary crystallographic information

### S1. Comment

The indazole core is recognized to be a highly effective pharmacophore in medicinal chemistry as well as being the core of important nitrogen-containing heterocycles that show a broad range of biological activities (Gaikwad *et al.*, 2015; Jennings & Tennant, 2007). Previously, our scientific team has pursued the research into derivatives of indazoles with the potential anticancer activity. We have synthesized and characterized indazoles bearing sulfonamide moieties. Some of them exert pharmacologically interesting antiproliferative/apoptotic activity against human and murine cell lines (Abbassi *et al.*, 2012; Abbassi *et al.*, 2014; Bouissane *et al.*, 2006).

The two fused five- and six-membered rings (N2,N3, C1–C7) of the indazole part of the molecule are almost planar, with a maximum deviation of 0.007 (2) Å at atom C1 (Fig. 1) and makes a dihedral angle of 74.99 (9)° with the mean plan trough the 4-methoxy-substituted benzene ring. The chloride atom and the sulfonamide group linked to the indazole ring are nearly coplanar with the largest deviation from the mean plane being 0.070 (2) Å at atom O1. The crystal structure exhibits inversion dimers in which molecules are linked by pairs of C15—H15C···O2 and N1—H1···O1 hydrogen bonds as shown in Fig. 2 and Table 1.

### S2. Experimental

A mixture of 3-chloro-6-nitroindazole (1.22 mmol) and anhydrous SnCl_2_ (1.1 g, 6.1 mmol) in absolute ethanol (25 ml) was heated at 333 K for 4 h. After reduction, the starting material reacted, and the solution was allowed to cool down. The pH was made slightly basic (pH 7–8) by the addition of 5% aqueous potassium bicarbonate before extraction with ethyl acetate. The organic phase was washed with brine and dried over magnesium sulfate. The solvent was removed to afford the amine, which was immediately dissolved in pyridine (5 ml) and then reacted with 4-methoxybenzenesulfonyl chloride (1.25 mmol) at room temperature for 24 h. After the reaction mixture was concentrated in vacuo, the resulting residue was purified by flash chromatography (eluted with ethyl acetate: hexane 1:9). The title compound was recrystallized from acetone (yield: 64%, m.p.: 388 K).

### S3. Refinement

H atoms were located in a difference map and treated as riding with C–H = 0.93–0.96 Å and N–H = 0.86 Å, and with with *U*_iso_(H) = 1.2 *U*_eq_ for aromatic–H and N–H and *U*_iso_(H) = 1.5 *U*_eq_ for methyl–H.

### Crystal data

**Table:**

C_16_H_14_ClN_3_O_4_S	*D*_x_ = 1.499 Mg m^−^^3^
*M**_r_* = 379.81	Melting point: 388 K
Monoclinic, *P*2_1_/*c*	Mo *K*α radiation, λ = 0.71073 Å
*a* = 13.9664 (6) Å	Cell parameters from 4461 reflections
*b* = 6.4300 (3) Å	θ = 3.0–29.0°
*c* = 19.6155 (9) Å	µ = 0.38 mm^−^^1^
β = 107.227 (1)°	*T* = 296 K
*V* = 1682.52 (13) Å^3^	Block, colourless
*Z* = 4	0.31 × 0.27 × 0.21 mm
*F*(000) = 784	

### Data collection

**Table:**

Bruker X8 APEX diffractometer	4461 independent reflections
Radiation source: fine-focus sealed tube	3423 reflections with *I* > 2σ(*I*)
Graphite monochromator	*R*_int_ = 0.032
φ and ω scans	θ_max_ = 29.0°, θ_min_ = 3.0°
Absorption correction: multi-scan (*SADABS*; Bruker, 2009)	*h* = −15→19
*T*_min_ = 0.654, *T*_max_ = 0.747	*k* = −8→8
33806 measured reflections	*l* = −26→26

### Refinement

**Table:**

Refinement on *F*^2^	0 restraints
Least-squares matrix: full	Hydrogen site location: inferred from neighbouring sites
*R*[*F*^2^ > 2σ(*F*^2^)] = 0.042	H-atom parameters constrained
*wR*(*F*^2^) = 0.130	*w* = 1/[σ^2^(*F*_o_^2^) + (0.0695*P*)^2^ + 0.2697*P*] where *P* = (*F*_o_^2^ + 2*F*_c_^2^)/3
*S* = 1.09	(Δ/σ)_max_ = 0.001
4461 reflections	Δρ_max_ = 0.34 e Å^−^^3^
227 parameters	Δρ_min_ = −0.35 e Å^−^^3^

### Special details

**Table:**

Geometry. All e.s.d.'s (except the e.s.d. in the dihedral angle between two l.s. planes) are estimated using the full covariance matrix. The cell e.s.d.'s are taken into account individually in the estimation of e.s.d.'s in distances, angles and torsion angles; correlations between e.s.d.'s in cell parameters are only used when they are defined by crystal symmetry. An approximate (isotropic) treatment of cell e.s.d.'s is used for estimating e.s.d.'s involving l.s. planes.

### Fractional atomic coordinates and isotropic or equivalent isotropic displacement parameters (Å^2^)

**Table:**

	*x*	*y*	*z*	*U*_iso_*/*U*_eq_	
C1	0.91669 (11)	0.2704 (2)	0.37321 (8)	0.0452 (3)	
C2	0.89374 (16)	0.1193 (3)	0.41556 (10)	0.0617 (5)	
H2	0.9006	−0.0205	0.4058	0.074*	
C3	0.86020 (16)	0.1769 (3)	0.47309 (10)	0.0602 (5)	
H3	0.8448	0.0755	0.5020	0.072*	
C4	0.85003 (11)	0.3830 (2)	0.48700 (8)	0.0408 (3)	
C5	0.87624 (15)	0.5333 (3)	0.44499 (10)	0.0550 (4)	
H5	0.8712	0.6734	0.4551	0.066*	
C6	0.90957 (15)	0.4760 (3)	0.38864 (10)	0.0557 (4)	
H6	0.9274	0.5773	0.3608	0.067*	
C7	0.61586 (12)	0.4460 (3)	0.47134 (9)	0.0473 (4)	
C8	0.53586 (11)	0.3118 (3)	0.44493 (8)	0.0441 (3)	
H8	0.5300	0.1886	0.4682	0.053*	
C9	0.46477 (11)	0.3708 (2)	0.38180 (8)	0.0446 (3)	
C10	0.47468 (13)	0.5525 (3)	0.34520 (9)	0.0516 (4)	
C11	0.55490 (14)	0.6856 (3)	0.37318 (12)	0.0617 (5)	
H11	0.5613	0.8077	0.3495	0.074*	
C12	0.62455 (14)	0.6340 (3)	0.43640 (11)	0.0601 (4)	
H12	0.6779	0.7234	0.4564	0.072*	
C13	0.39023 (14)	0.5496 (3)	0.28361 (10)	0.0573 (4)	
C14	0.32896 (13)	0.0995 (3)	0.35643 (9)	0.0507 (4)	
C15	0.22920 (16)	0.0485 (3)	0.30602 (11)	0.0669 (5)	
H15A	0.2340	0.0443	0.2582	0.100*	
H15B	0.1816	0.1529	0.3092	0.100*	
H15C	0.2076	−0.0846	0.3181	0.100*	
C16	0.95084 (18)	0.0198 (3)	0.29354 (11)	0.0681 (5)	
H16A	0.9737	0.0148	0.2520	0.102*	
H16B	0.9962	−0.0572	0.3315	0.102*	
H16C	0.8851	−0.0402	0.2826	0.102*	
N1	0.68799 (10)	0.3907 (3)	0.53612 (7)	0.0528 (3)	
H1	0.6685	0.3165	0.5660	0.063*	
N2	0.37631 (11)	0.2767 (2)	0.34114 (7)	0.0500 (3)	
N3	0.33132 (12)	0.3908 (3)	0.27965 (8)	0.0582 (4)	
O1	0.37051 (10)	−0.0002 (2)	0.40987 (7)	0.0588 (3)	
O2	0.85332 (9)	0.3360 (2)	0.61953 (6)	0.0641 (4)	
O3	0.81333 (11)	0.6802 (2)	0.56320 (8)	0.0681 (4)	
O4	0.94732 (10)	0.2289 (2)	0.31491 (7)	0.0593 (3)	
S1	0.80653 (3)	0.45883 (7)	0.55819 (2)	0.04892 (14)	
Cl1	0.36508 (5)	0.72884 (10)	0.21588 (3)	0.0859 (2)	

### Atomic displacement parameters (Å^2^)

**Table:**

	*U*^11^	*U*^22^	*U*^33^	*U*^12^	*U*^13^	*U*^23^
C1	0.0407 (8)	0.0515 (8)	0.0423 (8)	−0.0026 (6)	0.0104 (6)	0.0070 (6)
C2	0.0916 (14)	0.0368 (8)	0.0669 (11)	0.0011 (8)	0.0390 (10)	0.0044 (7)
C3	0.0858 (14)	0.0418 (8)	0.0645 (11)	−0.0039 (8)	0.0400 (10)	0.0088 (7)
C4	0.0355 (7)	0.0432 (7)	0.0427 (7)	−0.0029 (6)	0.0101 (6)	0.0004 (6)
C5	0.0692 (11)	0.0384 (8)	0.0621 (10)	−0.0086 (7)	0.0265 (9)	0.0016 (7)
C6	0.0703 (11)	0.0455 (8)	0.0564 (10)	−0.0113 (8)	0.0268 (9)	0.0078 (7)
C7	0.0397 (8)	0.0540 (9)	0.0529 (9)	0.0037 (6)	0.0209 (7)	−0.0007 (7)
C8	0.0424 (8)	0.0491 (8)	0.0444 (8)	0.0034 (6)	0.0183 (6)	0.0020 (6)
C9	0.0413 (8)	0.0500 (8)	0.0473 (8)	0.0052 (6)	0.0203 (6)	0.0007 (6)
C10	0.0481 (9)	0.0563 (9)	0.0561 (9)	0.0108 (7)	0.0242 (8)	0.0105 (7)
C11	0.0542 (10)	0.0554 (10)	0.0804 (13)	0.0032 (8)	0.0271 (9)	0.0182 (9)
C12	0.0471 (9)	0.0587 (10)	0.0758 (12)	−0.0038 (8)	0.0202 (9)	0.0073 (9)
C13	0.0558 (10)	0.0662 (11)	0.0544 (9)	0.0142 (8)	0.0231 (8)	0.0173 (8)
C14	0.0497 (9)	0.0546 (9)	0.0463 (8)	0.0004 (7)	0.0118 (7)	−0.0034 (7)
C15	0.0639 (12)	0.0710 (12)	0.0545 (10)	−0.0106 (9)	0.0002 (9)	−0.0021 (9)
C16	0.0777 (13)	0.0725 (12)	0.0595 (11)	0.0117 (10)	0.0288 (10)	−0.0048 (9)
N1	0.0420 (7)	0.0696 (9)	0.0487 (7)	−0.0057 (6)	0.0165 (6)	0.0042 (6)
N2	0.0498 (7)	0.0563 (8)	0.0420 (7)	0.0026 (6)	0.0107 (6)	0.0055 (6)
N3	0.0568 (9)	0.0696 (10)	0.0468 (8)	0.0097 (7)	0.0132 (7)	0.0115 (7)
O1	0.0532 (7)	0.0599 (7)	0.0572 (7)	−0.0037 (6)	0.0068 (6)	0.0094 (6)
O2	0.0522 (7)	0.0939 (10)	0.0416 (6)	−0.0115 (7)	0.0070 (5)	0.0031 (6)
O3	0.0687 (8)	0.0608 (8)	0.0779 (9)	−0.0131 (6)	0.0264 (7)	−0.0248 (7)
O4	0.0719 (8)	0.0625 (7)	0.0502 (7)	−0.0038 (6)	0.0286 (6)	0.0018 (5)
S1	0.0440 (2)	0.0576 (3)	0.0458 (2)	−0.00842 (17)	0.01419 (17)	−0.00832 (17)
Cl1	0.0821 (4)	0.0971 (4)	0.0790 (4)	0.0150 (3)	0.0247 (3)	0.0444 (3)

### Geometric parameters (Å, º)

**Table:**

C1—O4	1.361 (2)	C11—C12	1.371 (3)
C1—C6	1.366 (2)	C11—H11	0.9300
C1—C2	1.376 (2)	C12—H12	0.9300
C2—C3	1.394 (3)	C13—N3	1.299 (3)
C2—H2	0.9300	C13—Cl1	1.7146 (18)
C3—C4	1.369 (2)	C14—O1	1.219 (2)
C3—H3	0.9300	C14—N2	1.394 (2)
C4—C5	1.388 (2)	C14—C15	1.487 (2)
C4—S1	1.7490 (16)	C15—H15A	0.9600
C5—C6	1.371 (3)	C15—H15B	0.9600
C5—H5	0.9300	C15—H15C	0.9600
C6—H6	0.9300	C16—O4	1.414 (2)
C7—C8	1.385 (2)	C16—H16A	0.9600
C7—C12	1.413 (2)	C16—H16B	0.9600
C7—N1	1.413 (2)	C16—H16C	0.9600
C8—C9	1.391 (2)	N1—S1	1.6419 (14)
C8—H8	0.9300	N1—H1	0.8600
C9—N2	1.395 (2)	N2—N3	1.3924 (19)
C9—C10	1.399 (2)	O2—S1	1.4257 (14)
C10—C11	1.388 (3)	O3—S1	1.4279 (14)
C10—C13	1.417 (3)		
			
O4—C1—C6	115.95 (14)	C11—C12—H12	119.8
O4—C1—C2	123.80 (16)	C7—C12—H12	119.8
C6—C1—C2	120.25 (16)	N3—C13—C10	114.44 (15)
C1—C2—C3	119.71 (16)	N3—C13—Cl1	120.08 (15)
C1—C2—H2	120.1	C10—C13—Cl1	125.44 (15)
C3—C2—H2	120.1	O1—C14—N2	118.65 (15)
C4—C3—C2	119.87 (15)	O1—C14—C15	124.73 (17)
C4—C3—H3	120.1	N2—C14—C15	116.61 (15)
C2—C3—H3	120.1	C14—C15—H15A	109.5
C3—C4—C5	119.64 (15)	C14—C15—H15B	109.5
C3—C4—S1	120.68 (12)	H15A—C15—H15B	109.5
C5—C4—S1	119.68 (13)	C14—C15—H15C	109.5
C6—C5—C4	120.27 (16)	H15A—C15—H15C	109.5
C6—C5—H5	119.9	H15B—C15—H15C	109.5
C4—C5—H5	119.9	O4—C16—H16A	109.5
C1—C6—C5	120.20 (15)	O4—C16—H16B	109.5
C1—C6—H6	119.9	H16A—C16—H16B	109.5
C5—C6—H6	119.9	O4—C16—H16C	109.5
C8—C7—C12	121.82 (16)	H16A—C16—H16C	109.5
C8—C7—N1	117.46 (15)	H16B—C16—H16C	109.5
C12—C7—N1	120.70 (16)	C7—N1—S1	124.38 (12)
C7—C8—C9	116.57 (15)	C7—N1—H1	117.8
C7—C8—H8	121.7	S1—N1—H1	117.8
C9—C8—H8	121.7	N3—N2—C14	119.73 (14)
C8—C9—N2	131.97 (15)	N3—N2—C9	111.28 (14)
C8—C9—C10	122.10 (16)	C14—N2—C9	128.90 (13)
N2—C9—C10	105.92 (14)	C13—N3—N2	104.30 (15)
C11—C10—C9	120.21 (16)	C1—O4—C16	118.88 (14)
C11—C10—C13	135.73 (17)	O2—S1—O3	119.31 (9)
C9—C10—C13	104.05 (16)	O2—S1—N1	104.43 (8)
C12—C11—C10	118.81 (17)	O3—S1—N1	108.98 (9)
C12—C11—H11	120.6	O2—S1—C4	109.79 (8)
C10—C11—H11	120.6	O3—S1—C4	107.52 (8)
C11—C12—C7	120.40 (17)	N1—S1—C4	106.07 (7)

### Hydrogen-bond geometry (Å, º)

**Table:**

*D*—H···*A*	*D*—H	H···*A*	*D*···*A*	*D*—H···*A*
N1—H1···O1^i^	0.86	2.19	2.934 (2)	144
C15—H15*C*···O2^i^	0.96	2.33	3.251 (3)	159

Symmetry code: (i) −*x*+1, −*y*, −*z*+1.
