# Supplementary figures and images for: Crystal structure of N-(1-acetyl-3-chloro-1H-indazol-6-yl)-4-meth­oxy­benzene­sulfonamide
Source: Acta Crystallogr E Crystallogr Commun. 2015 Nov 4;71(Pt 12):o914–5. doi: 10.1107/S2056989015020605 (PMC4719877; doi:10.1107/S2056989015020605)

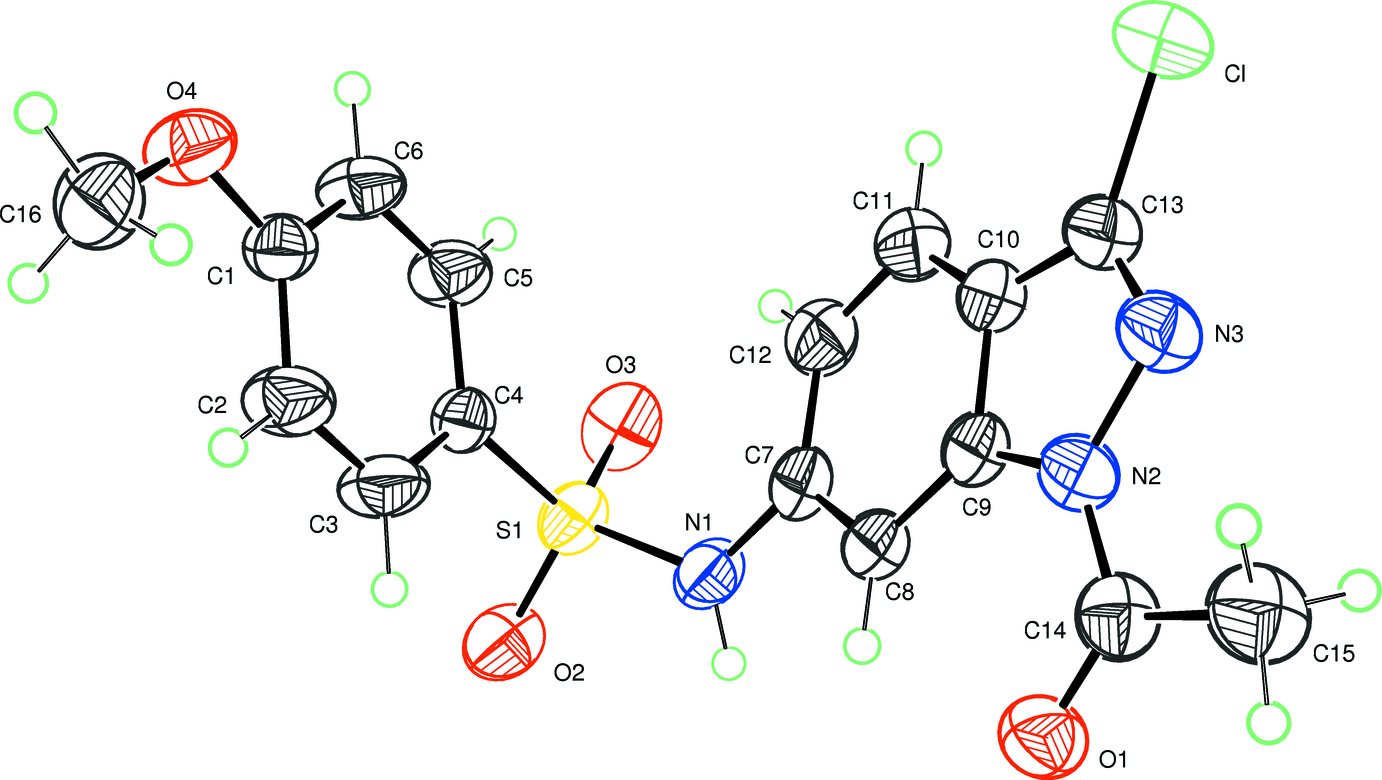

Supplement: Supplementary file 4 [file e-71-0o914-fig1.tif]

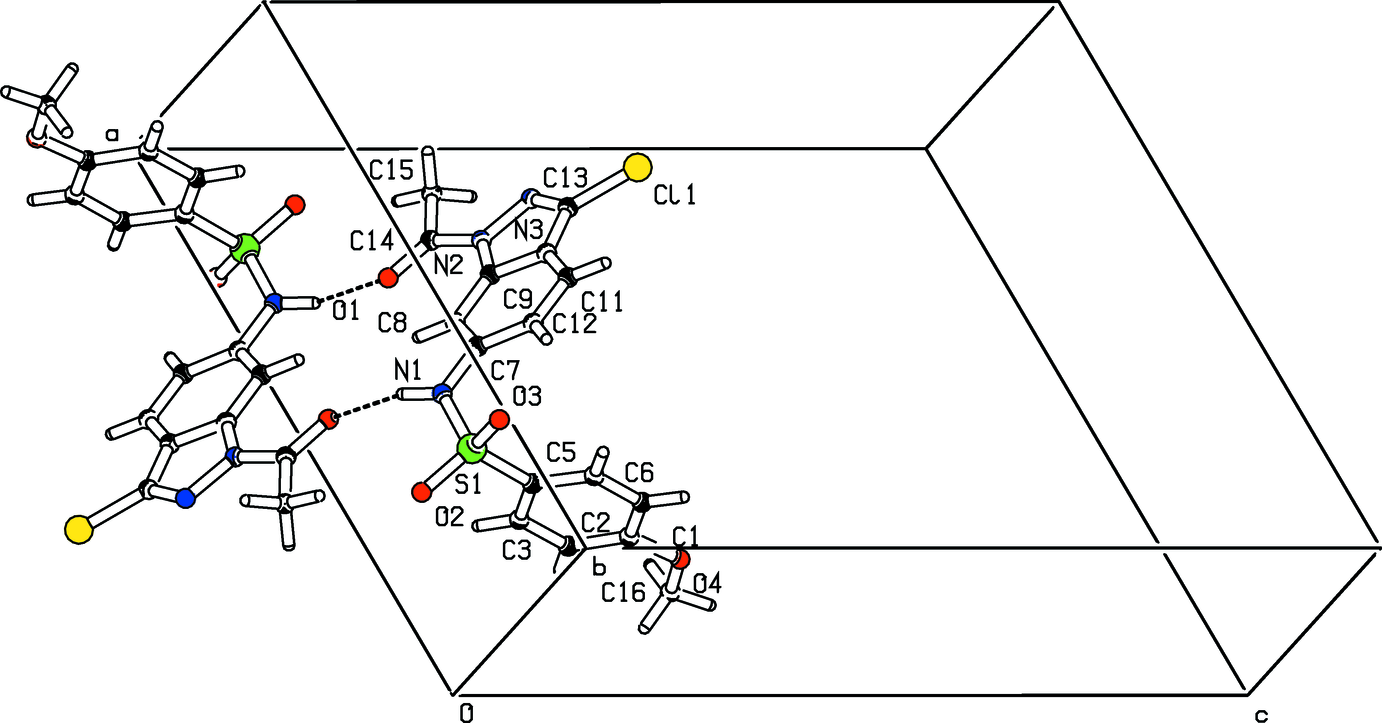

Supplement: Supplementary file 5 [file e-71-0o914-fig2.tif]
